# Glioblastoma multiforme: a multi-omics analysis of driver genes and tumour heterogeneity

**DOI:** 10.1098/rsfs.2020.0072

**Published:** 2021-06-11

**Authors:** Gabriel Emilio Herrera-Oropeza, Carla Angulo-Rojo, Santos Alberto Gástelum-López, Alfredo Varela-Echavarría, Maribel Hernández-Rosales, Katia Aviña-Padilla

**Affiliations:** ^1^ Instituto de Neurobiología, Universidad Nacional Autónoma de México, Querétaro, Mexico; ^2^ Centre for Developmental Neurobiology, Institute of Psychiatry, Psychology, and Neuroscience, King's College London, London, UK; ^3^ Centro de Investigación Aplicada a la Salud, Facultad de Medicina, Universidad Autónoma de Sinaloa, Culiacán, Sinaloa, Mexico; ^4^ Centro Interdisciplinario de Investigación para el Desarrollo Integral Regional, Instituto Politécnico Nacional, Guasave, Sinaloa, Mexico; ^5^ Centro de Investigación y de Estudios Avanzados del IPN, Unidad Irapuato, Guanajuato, Mexico

**Keywords:** glioma, cancer genomics, tumour heterogeneity, diagnosis, biomarkers

## Abstract

Glioblastoma (GBM) is the most aggressive and common brain cancer in adults with the lowest life expectancy. The current neuro-oncology practice has incorporated genes involved in key molecular events that drive GBM tumorigenesis as biomarkers to guide diagnosis and design treatment. This study summarizes findings describing the significant heterogeneity of GBM at the transcriptional and genomic levels, emphasizing 18 driver genes with clinical relevance. A pattern was identified fitting the stem cell model for GBM ontogenesis, with an upregulation profile for *MGMT* and downregulation for *ATRX, H3F3A, TP53* and *EGFR* in the mesenchymal subtype. We also detected overexpression of *EGFR, NES, VIM* and *TP53* in the classical subtype and of *MKi67* and *OLIG2* genes in the proneural subtype. Furthermore, we found a combination of the four biomarkers *EGFR, NES, OLIG2* and *VIM* with a remarkable differential expression pattern which confers them a strong potential to determine the GBM molecular subtype. A unique distribution of somatic mutations was found for the young and adult population, particularly for genes related to DNA repair and chromatin remodelling, highlighting *ATRX, MGMT* and *IDH1.* Our results also revealed that highly lesioned genes undergo differential regulation with particular biological pathways for young patients. This multi-omic analysis will help delineate future strategies related to the use of these molecular markers for clinical decision-making in the medical routine.

## 1. Introduction

Glioblastoma multiforme (GBM) is the most frequent and aggressive deadly primary brain tumour in adults, accounting for approximately 82% of all malignant gliomas [1]. Although it can affect children, its incidence rises with age. GBM tumours are characterized by increased cell proliferation, aggressive invasion, active angiogenesis and a remarkable genetic heterogeneity [2]. Histologically, tumours display a high morphological variability as they contain pleomorphic and multinucleated cells with high mitotic activity, show microvascular proliferation, undergo severe and characteristic endothelial hyperplasia, contain intravascular microthrombi, and extensive necrosis of an ischaemic or pseudo-empalized nature. The multiforme denomination of GBM tumours is due to the diverse and heterogeneous microenvironments that parallel their multiple histological patterns and cytological features.

According to their ontogeny, most GBMs are primary tumours that develop de novo in the absence of previous neoplasia. Primary GBMs are highly aggressive and invasive, tend to extend to both cerebral hemispheres, or are bilateral, and they are most commonly manifested in elderly patients. Secondary GBMs, in contrast, are located in the frontal lobe and develop mainly in younger patients suffering from anaplastic astrocytoma or low-grade astrocytoma, presenting a much better prognosis [3]. Recent reports have determined that primary and secondary glioblastomas have distinct genetic alterations related to particular biological pathways [1,3,4], suggesting they require different therapeutic approaches. Hence, from the clinical perspective, discerning between primary and secondary GBM is highly relevant [2]. Usually, primary GBMs present overexpression and gene amplification of epidermal growth factor receptor (*EGFR*) and mutations in cyclin-dependent kinase inhibitor 2A (*CDKN2A/p16INK4A*) and phosphatase tensin homologue (*PTEN*) genes. Molecular biomarkers of secondary GBM include mutations in tumour protein 53 (*TP53*) and isocitrate dehydrogenase-1 (*IDH1*) genes, which correlate strongly with O_6_-methylguanine-DNA methyltransferase (*MGMT*) promoter methylation [3,5].

Initiation and progression of GBM tumorigenesis are related to genetic and epigenetic alterations and molecular subtypes of GBM have unique transcriptional profiles. Based on expression features, GBM tumours were originally classified into four subtypes: neural, proneural, classical (proliferative) and mesenchymal [6], a scheme that has been recently revised using transcriptomic information. The improved classification eliminates the neural subtype and considers tumours of this molecular type as containing normal brain tissue contamination [7,8]. GBM molecular subtypes are also associated with different spatial zones, heterogeneity and aggressiveness of the tumour [9].

GBMs belonging to the proneural subtype have alterations in *TP53, PDGFRA, PIK3CA* and *IDH1* genes [10,11]. The classical subtype, also known as a proliferative subtype, has been associated with high levels of cell proliferation and upregulation of *EGFR* [12]. Mesenchymal GBMs show overexpression of mesenchymal and astrocytic markers (*CD44*, and *MERTK*) and downregulation of neurofibromatosis type-1 (NF1) and upregulation of chitinase 3 like 1 (*CHI3L1/YKL-40*) and *MET* are frequently observed [10]. While the proneural subtype has been mostly reported in younger patients and is associated with a favourable prognosis, the mesenchymal and the classical subtypes are usually linked to more aggressive high-grade gliomas that appear in adult or elderly life.

Recent advances employing next-generation sequencing have led to a better insight into the molecular biology of gliomas contributing potential markers for better diagnosis and new approaches to finding specific treatment strategies [13]. GBM remains an incurable deadly disease with an abysmal prognosis that has not significantly shown improvement, causing an enormous individual and societal burden. Thus, there is a need for tumour-specific drug targets and pharmacological agents to inhibit cell migration, dispersal and angiogenesis [7]. For a current detailed review, see [14].

In the last years, the clinical relevance of GBM heterogeneity has been highlighted [15]. This particular feature makes this type of cancer one of the most challenging to treat and consists of *inter-tumour* and *intra-tumour* feature variations. Inter-tumour heterogeneity refers to GBMs from different patients with altered and differing genotypes and phenotypes related to diverse etiological and environmental factors. On the other hand, intra-tumour heterogeneity refers to the presence of multiple and different cell subpopulations within the same tumour, defining its topology and architecture [16]. The comprehensive genomic classification of GBM paves the way for an improved understanding of tumour progression, which in the future may result in personalized therapy. Hence, there is an urgent need to further our knowledge of tumour heterogeneity as it will help design better therapies against GBM and tumour recurrence.

Based on a multi-omic analysis, in this study we describe the heterogeneity of GBM at the transcriptional and genomic levels, with emphasis on driver genes currently used as biomarkers. For that purpose, from 60 clinical reports, we selected and analysed 18 driver genes that have shown deregulated behaviour in patient samples. Using bioinformatics pipelines and the TCGA database, we examined their mRNA expression in the different GBM molecular subtypes and the presence of somatic mutations linked to possible disruption of protein function. We hope that the new knowledge generated in this study leads to novel therapeutic intervention strategies.

## 2. Material and methods

### 2.1. Data mining for selection of GBM driver genes currently used as genomic markers in the clinic

The literature research was performed using a systematic approach to identify GBM biomarkers in the routine clinical diagnosis that yielded differential transcriptomics or genomic profiles on tumour samples. Using a combination of three terms: (1) ‘*Glioblastoma’,* (2) *‘Clinical’ *and** (3) ‘*Case’*, a total of 3238 clinical reports were found using the BVS (1548), Cochrane (0), Karger (271) and PubMed (1419) databases. Clinical reports were identified and selected by title and summary. All articles were evaluated using the guidelines of the Preferred Reporting Items for Systematic Reviews and Meta-Analyses (PRISMA) (http://prisma-statement.org/) to determine their eligibility, resulting in 60 reports, as described in electronic supplementary material, file 1. The search was conducted in July 2020 and focused on studies published in June 2005–June 2020.

### 2.2. Data source for the gene expression analysis

Eighteen genes were found to be involved in GBM diagnosis during the neuro-oncology clinical routine and evaluated for their mRNA expression analysis using data from the Glioblastoma BioDiscovery Portal (GBM-BioDP) https://gbm-biodp.nci.nih.gov [17]. The gene expression data include normalized (level 3) data from Verhaak 840 Core, a filtered dataset conformed of three microarray platforms: HT_HG-U133A (488 patient samples/612 042 features), HuEx-1_0-st-v2 (437 patient samples/618 631 features), and AgilentG4502A_07_1/2 (101 + 396 patient samples/617 813 features). GBM molecular subtypes were assigned according to the Verhaak classification [11].

### 2.3. Determination of gene expression of GBM driver genes

We classified the mRNA expression analysis of the driver genes according to their biological ontology into three groups: (1) DNA repair and chromatin remodelling, (2) cytoskeleton and cellular proliferation, and (3) tumour suppressors genes. Using Python scripts (https://github.com/kap8416/GBM-META-ANALYSIS-OF-DRIVER-GENES), we determined the average and standard deviation of the *z*-score expression values for all patient results and classified them into the molecular GBM subtypes (Classic, Proneural, Mesenchymal), and grouped them by their corresponding biological gene ontology group.

We examined the mRNA expression patterns of the driver genes clustered by patient subgroups taking their age into account. From a total of 166 patients, three subgroups were created: 10–29-year-old patients (young subgroup), 30–59 years old (adult subgroup) and 60–89 years old (elderly subgroup). The average of the *z*-score values among the patient subgroups was clustered into the molecular GBM subtypes.

Finally, the Mann–Whitney test was used to examine the statistical difference in the mRNA expression *z*-scores between GBM molecular subtypes and patient subgroups and between each gene and GBM subtypes. Multiplicity adjustments were performed on the obtained *p*-values by using the Benjamini–Hochberg method. Statistical significance for the test was set to *p* < 0.05.

### 2.4. Data source for somatic mutations of GBM

Genomic data from 588 patients for the 18 genes previously identified as molecular markers was downloaded from the NIH website https://portal.gdc.cancer.gov/ using the following restriction criteria: Primary site: brain; Program: TCGA; Project: TCGA-GB; Disease Type: glioblastoma; Sample type: primary tumour; Clinical age of diagnosis: 10–29 years, 30–59 years and 60–89 years.

### 2.5. Determination of mutations in GBM and driver genes

Using Python scripts (https://github.com/kap8416/GBM-META-ANALYSIS-OF-DRIVER-GENES), the number of mutations per gene in the TCGA-GBM project was determined by calculating the amount of different genomic DNA changes reported in each gene. Subsequently, the relative percentage of mutations per chromosome was calculated by taking into account the total length (base pairs) of the respective chromosome. Substitutions, deletions and insertions were identified, then the number of nucleotide changes occurring in all genes was determined, and their distribution was compared to the distribution of those present in the driver genes. Moreover, the total number of mutations per gene and the genome location of the somatic mutations were compared among patient subgroups according to their age. Finally, the protein phenotype impact values (polyphen) of all the canonical missense variant consequences of the driver genes in the TCGA-GBM project were determined, analysed and compared between patient subgroups clustered by age.

### 2.6. Functional enrichment for driver genes, unique or shared pathways

GO enrichment analysis was performed using the Metascape tool (http://metascape.org/). We then used the meta-analysis workflow to compare the driver gene pathways with those of the highly mutated genes to identify unique or shared biological pathways in which they are involved. Using Python scripts, the top 50 mutated genes observed in the TCGA-GBM project were clustered by age group. Those genes were selected and analysed by their GO enriched terms. Finally, affected genes in their protein polymorphism phenotype with more than three probably damaging consequences (PR) were clustered by the patient subgroups for the GO terms and TRRUST enrichment analysis.

## 3. Results

### 3.1. Selection of the GBM driver genes as genomic markers in the clinic

First, we aimed to identify and select the top used biomarkers in the clinic. Sixty clinical reports were found from 2005 to 2020 that were fully text reviewed (described in electronic supplementary material, F1). A total of 73 patients with GBM were characterized and described in table 1. Patient demographics consisted of 43 men and 30 women with ages ranging from four to 78 years and a mean age of 43.31. Twenty-two patients were classified as young (4–29 years), 30 as adults (30–59 years) and 21 as elderly (60–78 years).

**Table 1.** Summary of selected clinical cases of GBM (n.a., Not/applicable).

reference^a^	database^b^	gender^c^	age^d^	symptoms^e^	type of gbm tumour^f^	surgical resection^g^	therapy^h^	tumour recurrence^i^	molecular markers (expression)^j^	molecular markers (somatic mutations)^k^	survival time^l^
Dormegny *et al.* [35]	BVS	male	21	hemiparesis, seizures	primary	n.a.	n.a.	yes	n.a.	H3K27M(+), IDH R132H(−)	3 months
Kajitani *et al.* [36]	BVS	male	13	headache	secondary	partial	QT/RT, TMZ	yes	GFAP(+),VIMENTIN(+),KI67(+),MGMT(−)	ATRX(+), IDH−1 wt(+), H3KM27M(−), BRAFV600E(−), TP53(+)	4 months
Kajitani *et al.* [36]	BVS	female	16	seizures	secondary	partial	QT/RT, TMZ	yes	GFAP(+), ATRX(+),VIMENTIN(+), KI67(+), MGMT(−)	IDH-1 wt (+), TP53(−), BRAFV600E(−),H3K27M(−)	6 months
Kajitani *et al.* [36]	BVS	female	16	facial nerve paralysis	secondary	total	QT/RT, TMZ	no	GFAP(+), ATRX(+), VIMENTIN(+),KI67(+)	IDH-1 wt (+),BRAFV600E(−),H3K27M(−), TP53(+)	alive
Kumaria *et al.* [37]	BVS	male	65	headache, personality disorder, dizziness	primary	total	n.a.	yes	GFAP(+)	IDH-1 wt(+)	17 months
McClelland *et al.* [38]	BVS	male	57	headache, hemiparesis	primary	partial	QT/RT, TTF	yes	GFAP(+),VIMENTIN (+), KI67(+)	TP53(+), IDH-1(+)	25 months
Petzold *et al.* [39]	BVS	female	28	headache, aphasia, dizziness, nausea	primary	total	QT/RT	no	n.a.	IDH-1(+)	alive
Prelaj *et al.* [40]	BVS	male	60	aphasia, hemiparesis	primary	total	QT/RT, TMZ	yes	GFAP(+), EGFR(+), MKi67(+)	TP53(+), IDH-1 wt(+)	6 months
Ranjan *et al.* [41]	BVS	female	51	headache, hemiparesis	primary	total	QT/RT,TMZ, nivolumab	no	KI67(+)	n.a.	alive
Ranjan *et al.* [41]	BVS	male	63	aphasia	primary	total	QT/RT,TMZ, nivolumab	no	KI67(+)	n.a.	alive
Ranjan *et al.* [41]	BVS	male	47	headache, nausea, vomiting	primary	total	QT/RT,TMZ, nivolumab, ipilimumab	yes	KI67(+)	n.a.	alive
Ranjan *et al.* [41]	BVS	male	47	headache, aphasia	primary	total	QT/RT,TMZ, nivolumab	yes	KI67(+)	n.a.	alive
Richard *et al.* [42]	BVS	male	28	seizures	primary	total	QT/RT,TMZ	no	GFAP(+), OLIG2(+), ATRX(+), MKi67(+), MGMT(+)	IDH-1(+),TP53(+), TERT(+)	alive
Rosen *et al.* [43]	BVS	female	48	aphasia, hemiparesis	primary	partial	QT/RT, TMZ, bevacizumab	yes	MGMT(−)	IDH-1 wt(+)	13 months
Wang *et al.* [44]	BVS	male	4	headache, hemiparesis, vomiting	primary	total	QT/RT, bevacizumab, nimotucimab, irinotecan	yes	GFAP(+),VIMENTIN(+),OLIG2(+), S-100(+)ATRX, MGMT(+),MKI67(+)	TP53, IDH-1(+), SMAD3(+), SMARCB1(+)	8 years
Bärtschi *et al.* [45]	BVS	male	44	hemiparesis	primary	total by 5-ALA fluorescence	QT/RT	no	S100A1(+),	BRAFV600E(−)	alive
Porto *et al.* [46]	BVS	male	72	headache	primary	partial by 5-ALA fluorescence	QT/RT	yes	ATRX(+)	IDH-1 wt(+)	5 months
Awadalla *et al.* [47]	BVS	male	60	aphasia, hemiplegia	primary	partial	n.a.	no	GFAP(+), VIMENTIN(+)	n.a.	8 months
Gestrich *et al.* [48]	BVS	male	64	altered mental status	primary	total	n.a.	yes	GFAP(+), S100(+)	IDH-1 wt(+)	10 months
Macchi *et al.* [49]	BVS	female	43	seizures, memory loss	primary	n.a.	QT/RT TMZ	no	n.a.	IDH-1 wt(+)	9 months
Watanabe *et al.* [50]	BVS	female	19	headache	secondary	total	QT/RT TMZ	yes	OLIG2(+), KI67(+), ATRX(−)	BRAFV600E(+), IDH1 R132H(−), SMARCB1(−), H3F3A(−) H3K27M(−), (TERT(−)	alive
Widjaja *et al.* [51]	Karger	male	58	hemiparesis, fever, progressive confusion	primary	total	QT/RT, Procarbacin	yes	GFAP(+), VIMENTIN(+)	n.a.	alive
Hou *et al.* [52]	Karger	female	30	aphasia, seizures	primary	partial	QT/RT TMZ	yes	S-100(+), GFAP(+), VIMENTIN(+)		5 months
Naydenov *et al.* [53]	Karger	female	45	headache, hemiparesis	primary	partial	QT/RT,TMZ	yes	EGRF(+)	TP53(+)	alive
Roviello *et al.* [54]	Karger	female	72	dizziness	primary	partial	QT/RT TMZ, corticostoroids	yes	MKi67(+), EGFR(−)	TP53(−)	4 months
Roviello *et al.* [54]	Karger	male	76	headache, hemiparesis	primary	partial	corticosteroids	no	MKi67(+), EGFR(+)	TP53(−)	5 months
Elzinga *et al.* [55]	Karger	female	76	hemiparesis, aphasia, confusion	primary	partial By CyberKnife	QT/RT, TMZ, bevacizumab	yes	OLIG2(+), MGMT(+)	IDH-1(−), EGFR(+)	22 months
Naydenov *et al.* [56]	Karger	male	61	aphasia, hemiparesis	secondary	partial	QT/RT	yes	MGMT(+)	n.a.	alive
Lewis *et al.* [57]	Karger	female	47	headache, nausea, hemiparesis	primary	total	QT/RT, TMZ, IFN-β	yes	GFAP(+), TP53(+), MGMT(−)	IDH-1 wt(+)	5 months
Papaevangelou *et al.* [58]	Karger	female	7	hemiparesis, physical disability	primary	total	QT/RT, temozolomide, erlotinib	yes	GFAP(+), S-100(+), VIMENTIN(+), OLIG2(+), MKI67(+),	EGFR(+), SMARCB1(+), H3K27M(+), SMAD3(−), TP53(−)	20 months
Hasan *et al.* [59]	Karger	female	58	hemiparesis	secondary	total	QT/RT	no	MKI67 (+), MGMT(−)	IDH-1 wt(+)	alive
Van Seggelen *et al.* [60]	Karger	male	62	ataxia	primary	total	QT/RT TMZ, nivolumab	yes	MGMT(+)	IDH-1 wt(+)	alive
Thummalapalli *et al.* [61]	Karger	male	74	aphasia	primary	partial	QT/RT, TMZ, nivolumab	yes	MGMT(−)	BRAFV600E(+), IDH-1 wt(+)	14 months
Rajagopalan *et al.* [62]	Pubmed	male	60	headache, hemiparesis	primary	partial	QT/RT TMZ, irinotecan, celecoxib	yes	GFAP(+)	n.a.	21 months
Zhang *et al.* [63]	PubMed	male	17	dysphagia, hypokinesia	primary	partial	QT TMZ	yes	GFAP(+), S100A1(+), VIMENTIN(+), MGMT(−), MKI67(−)	TP53(+), EGFR(−)	37 months
Zuccoli *et al.* [64]	PubMed	female	65	headache, nausea, memory loss	primary	partial	QT/RT TMZ irinotecan, bevacizumab	yes	MGMT(+)	n.a.	alive
Miao-Xia He *et al.* [65]	PubMed	male	31	headache	primary	total	QT/RT	yes	GFAP(+), S100A1(+), Vimentin(+), MKI67(+)	SMARCB1(+), SMA(+), TP53(+)	4 months
Paraskevopoulos *et al.* [66]	PubMed	female	12	hemiparesis, dysesthesia	primary	total	QT/RT vincristine, etoposide, carboplatin	yes	GFAP(+), S100A1(+), MKi67(+),	n.a.	12 months
Jeong *et al.* [67]	PubMed	male	32	headache	primary	total	QT/RT TMZ	no	GFAP(+), MKI67(+), MGMT(−)	EGFR(−)	alive
Lakičević *et al.* [68]	Pubmed	male	53	headaches, nausea, vomiting	primary	total	QT/RT TMZ	no	GFAP(+)	n.a.	alive
Matsuda *et al.* [69]	Pubmed	male	69	facial pain	primary	partial	QT/RT TMZ	no	GFAP (+), MKI67(+),	EGFR(+), TP53(−), IDH-1 R132H(−)	alive
Theeler *et al.* [70]	PubMed	female	36	progressive neurologic deficits	secondary	n.a.	QT/RT TMZ	yes	n.a.	IDH1 wt R132H(+), BRAFV600E(+)	alive
Theeler *et al.* [70]	PubMed	male	32	progressive neurologic deficits	primary	partial	QT/RT TMZ, erlotinb	yes	PIK3CA(+)	IDH wt R132H(+)	alive
Johnson *et al.* [71]	PubMed	male	73	hemiparesis, seizures	primary	total	QT/RT TMZ	yes	MGMT(+)	n.a.	24 months
Johansen *et al.* [72]	PubMed	female	59	headache, blurred vision	primary	total	QT/RT TMZ, bevacizumab	no	GFAP(+), OLIG2(+), MGMT(+), KI67(+), ATRX(+),	IDH-1(−), TP53(+)	8 months
Johansen *et al.* [72]	PubMed	male	60	seizures, cerebral haemorrhage	primary	total	n.a.	no	GFAP(+), OLIG2(+), MKI67(+), MGMT(+), ATRX(−)	IDH-1(−), TP53(−)	10 months
Anghileri *et al.* [73]	PubMed	male	43	headache	primary	total	RT/QT, TMZ, bevacizumab	yes	GFAP(+), VIMENTIN(+), MGMT(−)	EGFR(−)	25 months
Elena *et al.* [73]	PubMed	male	30	seizures	primary	total	QT/RT, TMZ, bevacizumab	yes	GFAP(+), VIMENTIN(+), MGMT(−)	IDH-1(−), EGFR(−)	6 years
Chen *et al.* [74]	PubMed	female	5	fever, vomiting	primary	total	n.a.	yes	MGMT(+), S100A1(+), GFAP(+), MKI67(+),	IDH-1 wt(−), TP53(+)	2 months
Gandhi *et al.* [75]	PubMed	female	45	aphasia	primary	partial	QT/RT	yes	MKI67(+)	TP53(+), EGFR(+), TERT(+), IDH-1 wt (−)	26 months
Efferth *et al.* [76]	PubMed	male	65	headache, seizures	primary	partial	QT/RT TMZ	no	MGMT(+)	n.a.	alive
Shen *et al.* [77]	PubMed	female	15	hemiparesis	primary	partial	QT/RT, TMZ	no	GFAP(+), KI67(+)	n.a.	13 months
Tokuda *et al.* [78]	PubMed	male	27	seizures, headache	secondary	total	QT/RT, TMZ, bevacizumab	yes	MKI67(+), VEGFR/FLT1(+)	IDH-1 Mutant(+)	alive
Wang *et al.* [79]	PubMed	female	50	headache, hemiparesis, nausea, vomiting	primary	total	RT	no	VIMENTIN(+), GFAP(+), OLIG2,(+), NESTIN(+)	IDH1-R132H(−), TP53(+), BRAFV600E(+)	alive
Wang *et al.* [79]	PubMed	male	36	headache, nausea, vomiting	primary	total	QT/RT, TMZ	yes	VIMENTIN(+), GFAP(+), OLIG2(+), NESTIN(+),	IDH1-R132H(−), TP53(−), BRAFV600E(+)	8 months
Zhang *et al.* [80]	PubMed	male	40	headache, hemiparesis, vomiting	primary	total	RT, TMZ	yes	KI-67(+), MGMT(−)	TP53(+)	alive
Zhou *et al.* [81]	PubMed	male	31	headache, vomiting	primary	total	QT/RT, TMZ	yes	GFAP(+), VIMENTIN(+), NESTIN(+), OLIG2(+), MKi67(+)	EGFR(+)	15 months
Comito *et al.* [82]	PubMed	female	57	headache, nausea, photopsia	primary	total	QT/RT, TMZ, lomustine n.a., nivolumab	yes	MKI67(+), GFAP(+), MGMT(+)	IDH-1 wt(−)	5 months
Finneran *et al.* [83]	PubMed	female	29	aphasia, headache, confusion	secondary	total	RT	no	GFAP(+), MGMT(−), S−100(−)	EGFR(−), SMARCB1(−), TP53(+), IDH-1-wt(−), BRAFV600E(−)	alive
Homma *et al.* [84]	PubMed	female	78	speech difficulty and forgetfulness	primary	partial	QT/RT, TMZ	no	S-100A1(+), GFAP(+), OLIG2(+), ATRX(+), MKI67(+),	SMARCB1(+), BRAFV600E(−), IDH-1-R132H(−)	alive
Janik *et al.* [85]	PubMed	male	51	headache, memory loss	primary	total	QT/RT, TMZ	yes	GFAP(+), MKi67(+)	TP53(+), BRAFV600E(+), IDH-1 wt (+), EGFR(+)	23 months
Narasimhaiah *et al.* [86]	PubMed	male	16	headache, vomiting, diplopia	primary	partial	QT/RT	yes	S-100A1(+), GFAP(+), MKi67(+), ATRX(−)	TP53(+), IDH-1(−)	alive
Narasimhaiah *et al.* [86]	PubMed	female	21	headache, seizures	primary	total	QT/RT	no	GFAP(+), MKi67(+), S100(+), ATRX(−)	TP53(+), IDH-1 R132H-mutant(−)	alive
Nørøxe *et al.* [87]	PubMed	male	62	confusion, aphasia	primary	partial	QT/RT, bevacizumab, irinotecan	yes	ATRX(−), MGMT(−)	IDH-1 wt(+)	15 months
Nørøxe *et al.* [87]	PubMed	male	30	headache, seizures, confusion	secondary	partial	QT/RT, TMZ	yes	GFAP(+), ATRX(+), MGMT(+)	IDH-1(+)	12 months
Chanchotisatien *et al.* [88]	PubMed	female	27	hemiparesis, dysuria	primary	partial	QT/RT, TMZ	no	GFAP(+), KI67(+), OLIG2(+), ATRX(+), Nestin(+),	H3K27M(+)	alive
Cuoco *et al.* [89]	PubMed	male	76	hemiparesis, clumsiness	primary	partial	QT/RT	no	MGMT(+),, EGFR(−),	IDH-1 wt (+), TP53(−)	1 months
Romo *et al.* [90]	PubMed	male	28	headache, nausea, personality changes, aphasia	primary	total	QT/RT, TMZ, VPC	yes	GFAP(+), OLIG2(+), ATRX(+), MGMT(+), S100(+)	IDH-1 mutant(+), TP53 mutant(+), SMARCB1(+), H3KM27(−)	3 months
Uppar *et al.* [91]	PubMed	female	28	hemiparesis	primary	total	n.a.	yes	GFAP(+), MKI67(+)	H3K27M(+), IDH-1 wt(−)	1 month
Woo *et al.* [92]	PubMed	female	22	headache	primary	total	RT, dabrafenib, trametinib	yes	MGMT(+)	BRAFV600E(+), IDH-1 wt(+)	7 months
Woo *et al.* [92]	PubMed	male	22	headache	primary	partial	BRAFi, vemurafenib, cobimetinib, palpociclib	yes	MGMT(+)	IDH-1 wt(+), BRAFV600E(+), TERT(+), EGFR(−)	8 months
Sajan *et al.* [93]	PubMed	female	39	headache	primary	n.a.	QT/RT TMZ	no	GFAP(+), MGMT(+)	EGFR(+), IDH-1wt(+), H3K27M(+), BRAFV600E(−)	alive
Gupta *et al.* [94]	PubMed	male	58	seizures	primary	total by 5-ALA fluorescence	QT/RT	yes	n.a.	IDH-1 wt R132H(+)	alive

^a^Reference of the clinical report.

^b^Database where the clinical report was found.

^c^Sex of the patient of the clinical case reported.

^d^Age of the patient of the clinical case reported.

^e^Symptoms described during clinical routine.

^f^GBM tumour according to ontogenesis subtypes.

^g^Surgical procedures during patient treatment.

^h^Medical and drugs administrated for treatment.

^i^Recurrence of tumour after surgical procedures.

^j^Gene expression measured for diagnosis.

^k^Molecular markers to identify somatic mutations.

^l^Survival time of patients after surgery and therapy treatment.

Patients underwent a biopsy procedure to evaluate the expression and mutations of biomarkers, which were the most representative genes used in clinical cases over the last 15 years. More than 80% of the clinical cases highlighted the use of a combination of 2–11 of the 18 markers. The most-reported were *IDH1, GFAP, MKi67* and *MGMT*, followed by *TP53, ATRX* and *EGFR*.

In this systematic review, only the biomarkers with differential positive results for patient diagnosis in the clinical reports were selected for further analysis (table 1). According to their Biological Process Gene Ontology, driver genes were clustered using k-means into three groups to determine their possible role in common pathways. The first group includes *ATRX, H3F3A, IDH1, MGTM* and *TERT* driver genes related to DNA repair and involved in chromatin remodelling pathways. The second group includes the cytoskeleton and cellular proliferation-related genes *EGFR, FLT1/(VEGFR), BRAF, GFAP, MKi67, NES, OLIG2, PIK3CA, SMAD3, S1001A* and *VIM.* In particular, *EGFR* has an essential role in activating the receptor tyrosine *kinase/Ras/phosphoinositide3-kinase RTK/RAS/PI3 K* pathway. Alterations in this pathway disrupt the G1-S transition in the cell cycle, which is highly relevant in the progression and excessive proliferation of GBM tumour cells. The third group included tumour suppressor genes *SMARCB1/INI1* and *TP53* which are negative regulators of cell growth control, normally acting to inhibit tumour development.

### 3.2. Transcriptomics analysis of driver genes of GBM tumorigenesis

Due to the high inter- and intra-tumour heterogeneity in GBM and to gain insight into this complex process, the expression profiling pattern of the top 18 genes used as biomarkers in the clinical report systematic review was analysed using gene expression data from the Glioblastoma BioDiscovery Portal. We focused on this analysis according to the Verhaak molecular classification of GBM, which groups tumours as proneural, classical and mesenchymal [11,16]. The gene expression analysis included all data available from the GBM-BioDP, including a total of 166 patients, from which 56 were proneural, 53 classical and 57 mesenchymal subtypes. Gene expression data from each patient were available for the 18 driver genes analysed (table 2).

**Table 2.** Summary of driver gene expression in GBM molecular subtypes with significant *p*-value. Data represent mean ± standard deviation for *z*-score in each gene. Statistical significance is represented by asterisks.

	proneural	classical	mesenchymal
DNA repair and chromatin remodelling genes			
ATRX	0.370 ± 0.936	0.084** ± 0.601	−0.213**** ± 0.591
BRAF	−0.152 ± 0.495	−0.237 ± 0.563	0.056 ± 0.468
H3F3A	0.340 ± 0.572	−0.079**** ± 0.613	−0.495**** ± 0.606
MGMT	−0.128 ± 1.237	−0.078 ± 1.464	0.614** ± 1.268
TERT	0.148 ± 0.385	0.26 ± 0.482	0.156 ± 0.490
cytoskeleton and cellular proliferation genes			
EGFR	−3.494 ± 3.780	3.502**** ± 4.360	−2.002* ± 3.787
FLT1	−0.571 ± 0.813	−0.301 ± 1.023	0.082** ± 1.093
GFAP	0.114 ± 0.870	0.367 ± 0.493	−0.293* ± 1.037
IDH1	−0.175 ± 0.881	0.484** ± 1.089	−0.168 ± 0.872
MKI67	1.019 ± 1.545	−0.114**** ± 1.269	−0.325**** ± 1.005
NES	−0.032 ± 0.852	1.525**** ± 1.004	0.053 ± 0.909
OLIG2	1.316 ± 1.182	0.070**** ± 1.173	−1.455**** ± 0.964
PIK3CA	0.241 ± 1.043	−0.178* ± 0.924	−0.146* ± 0.763
S100A1	0.520 ± 1.218	−0.723**** ± 1.063	−0.013* ± 1.464
SMAD3	−0.234 ± 0.711	0.300**** ± 0.425	0.261**** ± 0.579
VIM	−0.602 ± 1.134	0.805**** ± 0.973	0.671**** ± 0.878
Tumour suppressor genes			
SMARCB1	0.934 ± 0.884	0.425* ± 1.005	−0.393**** ± 0.893
TP53	0.101 ± 1.026	0.703*** ± 0.813	0.074 ± 0.775

*p < 0.05, **p < 0.01, ***p < 0.001 and ****p < 0.00001.

First, we analysed the overall profile expression pattern of each gene among GBM subtypes (table 2). For the DNA repair and chromatin remodelling genes, such as *ATRX* and *H3F3A*, we observed a tendency to a lower expression level in mesenchymal and an increased expression in proneural compared to the classic subtype. An inverse pattern was observed for *MGMT* with a tendency to be upregulated in mesenchymal and downregulated in proneural subtypes. Related to *TERT*, no expression differences are observed between the subtypes.

Among the cytoskeleton and cellular proliferation genes, the most substantial differences among subtypes are for *EGFR*, with a general tendency to be upregulated in the classical proliferative subtype and downregulated in the proneural and mesenchymal subtypes (table 2). Another tyrosine kinase growth factor, *FLT1*, did not show big differences in expression among GBM subtypes; meanwhile, *IDH1* has an upregulation tendency only for the classical subtype. The downstream effectors for growth factors, *PIK3CA* and *SMAD3*, showed upregulation and downregulation, respectively, for the proneural subtype; meanwhile, the pattern is inverse, downregulation for *PIK3CA* and upregulation for *SMAD3* for both the classical and mesenchymal subtypes (table 2). Another proliferation biomarker, *MKi67,* showed a marked overexpression in the proneural subtype and a tendency to downregulation in the classical and mesenchymal subtypes. *NES* and *VIM* appeared to be expressed more in the classic subtype than in other subtypes. Moreover, no relevant changes were observed for *GFAP,* another intermediate filament expressed in neural stem cells. Nevertheless, *OLIG2,* an oligodendrocyte marker, is upregulated in the proneural and downregulated in the mesenchymal subtype. The same behaviour was observed for the differentiation marker *S100A1* (table 2).

For the tumour suppressor genes, *TP53* is clearly upregulated in the proliferative classical subtype. The other gene, *SMARCB1,* is also overexpressed in the proneural and classical, but downregulated in the mesenchymal subtype (table 2).

We then analysed the expression patterns of the driver genes clustered into three subgroups of patients according to their age (tables 3–5). An important observation is that among tumours showing expression of these genes in patients under 30 years, the mesenchymal subtype was not observed (table 3). On the other hand, the driver gene expression in the mesenchymal subtype is only present in patients older than 80 years (data not shown).

**Table 3.** Summary of driver gene expression in GBM molecular subtypes in the 10–29 year subgroup with significant *p*-value. Data represent mean ± standard deviation for z-score in each gene. Statistical significance is represented by asterisks.

young subgroup (10–29 years)	proneural	classical
DNA repair and chromatin remodelling genes		
ATRX	0.105 ± 1.316	−0.105 ± 0.711
BRAF	0.257 ± 0.466	0.034 ± 0.777
H3F3A	0.375 ± 0.626	−0.202 ± 0.619
MGMT	0.297 ± 0.494	0.176 ± 1.782
TERT	0.066 ± 0.335	0.196 ± 0.464
cytoskeleton and cellular proliferation genes		
EGFR	−3.133 ± 1.228	−4.563 ± 2.382
FLT1	−0.946 ± 0.61	−0.955 ± 0.539
GFAP	−0.106 ± 0.914	0.262 ± 0.113
IDH1	−0.679 ± −0.679	−0.910 ± 0.204
MKI67	0.820 ± 2.157	0.572 ± 1.801
NES	−0.207 ± 1.018	0.822 ± 1.167
OLIG2	0.998 ± 1.427	−1.341* ± 0.859
PIK3CA	0.058 ± 0.526	0.272 ± 0.844
S100A1	0.452 ± 1.032	0.101 ± 0.807
SMAD3	0.104 ± 0.732	0.664 ± 0.438
VIM	−1.127 ± 1.343	1.455* ± 0.353
tumour suppressor genes		
SMARCB1	0.830 ± 0.653	0.653 ± 0.874
TP53	0.286 ± 0.993	−0.336 ± 1.242

*p < 0.05, **p < 0.01 and ***p < 0.001.

**Table 4.** Summary of driver gene expression in GBM molecular subtypes in the 30–59 year subgroup with significant *p*-value. Data represent mean ± standard deviation for *z*-score in each gene. Statistical significance is represented by asterisks.

adult subgroup (30–59 years)	proneural	classical	mesenchymal
DNA repair and chromatin remodelling genes			
ATRX	0.279 ± 0.920	0.113 ± 0.608	−0.180** ± 0.583
BRAF	−0.125 ± 0.392	−0.122 ± 0.561	0.064 ± 0.505
H3F3A	0.375 ± 0.446	−0.121**** ± 0.609	−0.451**** ± 0.602
MGMT	−0.354 ± 1.236	0.200 ± 1.339	0.786** ± 1.272
TERT	0.099 ± 0.402	0.357 ± 0.472	0.138 ± 0.451
cytoskeleton and cellular proliferation genes			
EGFR	−2.693 ± 3.863	3.314**** ± 4.265	−1.970 ± 3.678
FLT1	−0.637 ± 0.735	−0.259 ± 1.091	0.194* ± 1.220
GFAP	0.055 ± 1.018	0.249 ± 0.541	−0.399 ± 1.054
IDH1	−0.054 ± 0.833	0.781** ± 1.065	−0.115 ± 0.889
MKI67	1.065 ± 1.551	−0.052** ± 1.278	−0.061** ± 0.938
NES	−0.198 ± 0.700	1.380**** ± 0.884	−0.151 ± 0.941
OLIG2	1.396 ± 1.021	−0.029**** ± 1.083	−1.581**** ± 1.063
PIK3CA	0.415 ± 1.229	−0.370* ± 0.785	−0.328* ± 0.625
S100A1	0.467 ± 1.287	−0.674** ± 0.930	0.127 ± 1.516
SMAD3	−0.207 ± 0.838	0.271 ± 0.431	0.198 ± 0.542
VIM	−0.369 ± 1.083	0.564* ± 0.850	0.778** ± 0.944
tumour suppressor genes			
SMARCB1	1.008 ± 0.951	0.311* ± 0.998	−0.297**** ± 0.862
TP53	0.194 ± 1.162	0.829* ± 0.654	−0.124 ± 0.809

*p < 0.05, **p < 0.01, ***p < 0.001 and ****p < 0.00001.

**Table 5.** Summary of driver gene expression in GBM molecular subtypes in the 60–89 year subgroup with significant *p*-value. Data represent mean ± standard deviation for *z*-score in each gene. Statistical significance is represented by asterisks.

elderly subgroup (60–89 years)	proneural	classical	mesenchymal
DNA repair and chromatin remodelling genes			
ATRX	0.557 ± 0.718	0.077** ± 0.557	−0.255**** ± 0.597
BRAF	−0.331 ± 0.498	−0.474 ± 0.408	0.047* ± 0.416
H3F3A	0.293 ± 0.649	0.014 ± 0.606	−0.551**** ± 0.606
MGMT	−0.07 ± 1.377	−0.57 ± 1.449	0.394 ± 1.230
TERT	0.226 ± 0.372	0.121 ± 0.463	0.178 ± 0.535
cytoskeleton and cellular proliferation genes			
EGFR	−4.398 ± 4.122	5.495**** ± 2.32	−2.045** ± 3.921
FLT1	−0.368 ± 0.887	−0.229 ± 0.939	−0.062 ± 0.884
GFAP	0.253 ± 0.647	0.575 ± 0.382	−0.157 ± 0.997
IDH1	−0.103 ± 0.916	0.307 ± 0.966	−0.237 ± 0.844
MKI67	1.05 ± 1.226	−0.356*** ± 1.032	−0.663**** ± 0.987
NES	0.193 ± 0.864	1.904**** ± 1.004	0.314 ± 0.792
OLIG2	1.36 ± 1.204	0.524* ± 1.091	−1.293**** ± 0.792
PIK3CA	0.143 ± 0.968	0.03 ± 1.056	0.086 ± 0.855
S100A1	0.596 ± 1.211	−0.973*** ± 1.2	−0.192* ± 1.373
SMAD3	−0.387 ± 0.489	0.269**** ± 0.376	0.341**** ± 0.614
VIM	−0.629 ± 1.021	1.048**** ± 1.111	0.535*** ± 0.764
tumour suppressor genes			
SMARCB1	0.901 ± 0.886	0.602 ± 1.016	−0.515**** ± 0.916
TP53	−0.058 ± 0.864	0.724** ± 0.767	0.327 ± 0.645

*p < 0.05, **p < 0.01, ***p < 0.001 and ****p < 0.00001.

For the young subgroup, the samples were determined to belong only to proneural and classical subtypes, and from the 18 genes analysed, only *OLIG2* and *VIM* showed a differential pattern in gene expression. *OLIG2* is upregulated in the proneural tumours, according to its role as a differentiation biomarker. Meanwhile, our analysis revealed a downregulation tendency in the classical subtype. An inverse pattern was observed for *VIM,* which is downregulated in proneural and upregulated in the classical subtype. Interestingly, *EGFR* is downregulated in both subtypes (table 3).

Among the subgroup of adult patients, the behaviour of the *EGFR* stands out as it is upregulated in the classic subtype and downregulated in proneural and mesenchymal subtypes (table 4). The same pattern was observed in the elderly subgroup, but with a larger gap between subtypes (table 5). Analyses of genes *ATRX, H3F3A, MGMT, MKi67, NES, OLIG2, S100A1, VIM, SMARCB1* and *TP53* in the adult and elderly patients (tables 4 and 5) revealed the same pattern in the expression changes among subtypes as observed in the overall analysis (table 2).

**Figure 1.**
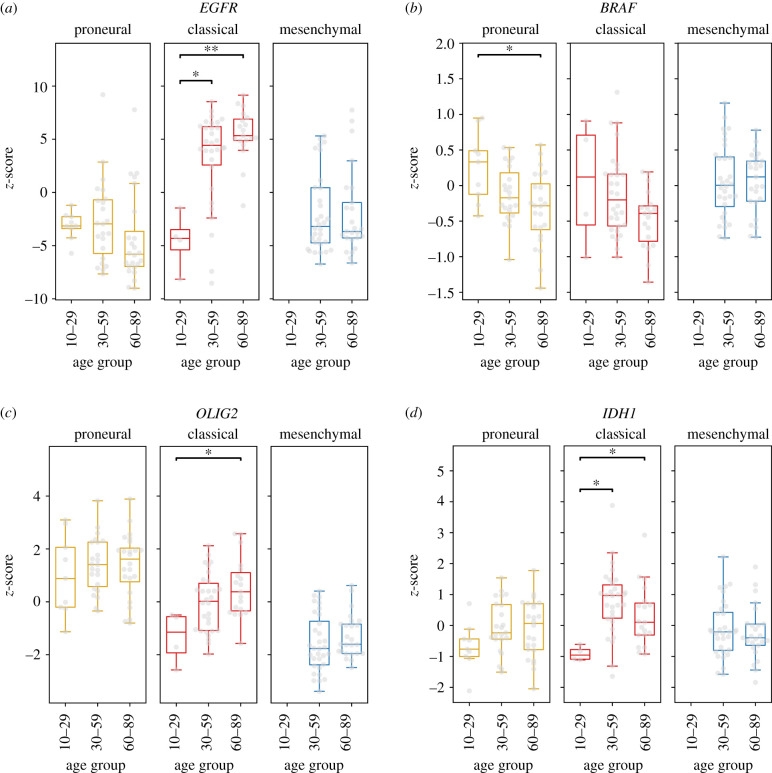
Comparison of driver gene expression profiles among patients grouped by GBM subtype. Gene expression data represented by *z*-score obtained from the Glioblastoma BioDiscovery Portal for driver genes, GBM subtype and patient age as indicated in each panel. Boxplot represents mean ± standard deviation. Statistical significance is represented by *p < 0.05, and **p < 0.01. Gene expression data from 13 patients. (M = 0; C = 4; P = 9) belonging to the age group of 10–29, 85 (M = 32, C = 30, P = 23) from the age group of 30–59, and 68 (M = 25, C = 19, P = 24) from the age group 60–89 was used. (mesenchymal = M, classical = C, proneural = P).

To analyse the variation of these biomarkers at different stages of life in each subtype, we selected the genes with the most remarkable differential expression pattern. The most common GBM biomarker, *EGFR* gene, showed a remarkable upregulation in the classic subtype from adult to elderly subgroups, while it was downregulated in the young subgroup. No differential pattern among ages was observed for the proneural or mesenchymal subtypes (figure 1*a*). For *BRAF,* a differential pattern was observed only in the proneural subtype, being upregulated in tumours from young patients and downregulated in elderly patients (figure 1*b*). *OLIG2* had a remarkable differential pattern in the classical subtype, in which it is downregulated in young patients and shows an upregulation in elderly patients (figure 1*c*). *IDH1* expression varies in the classical subtype, being downregulated in young patients and upregulated in both adult and elderly patients (figure 1*d*).

Summarizing, the gene expression analysis showed that the altered expression pattern in the mesenchymal subtype includes overexpression of *MGMT* that contributes to mutation development and downregulation of the differentiation biomarker *OLIG2* but upregulation of the stemness biomarker *VIM.* The altered expression profile in the classical subtype includes overexpression of the proliferation biomarker *EGFR* and the stemness biomarkers *NES* and *VIM.* The expression profile in the proneural subtype showed more characteristics of neural progenitor with the upregulation of *OLIG2*.

### 3.3. Somatic mutation analysis on driver genes

Gene mutation profiling has also served as a biomarker for the diagnosis and treatment of GBM. We used high-throughput data from the TCGA-GBM project and obtained the genomic profiles of a total of 588 clinical GBM cases.

**Figure 2.**
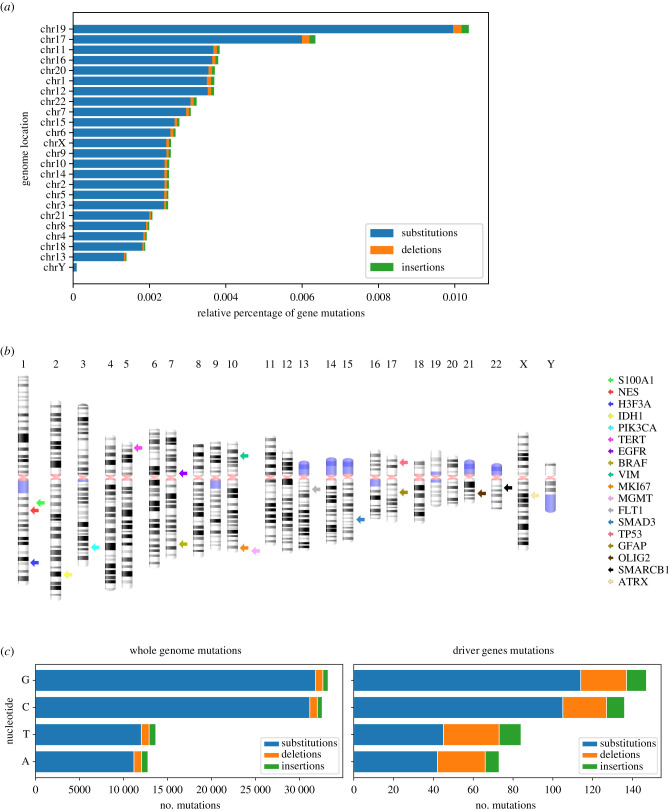
Distribution of the percentage of mutations in genes per chromosome observed in the TCGA-GBM project and the location of their mutations. (*a*) Relative percentage of gene mutations per chromosome shown by mutation type: substitutions (blue), deletions (orange) and insertions (green). (*b*) Cytogenetic representation of human chromosomes, rendered with standard banding patterns, showing the chromosomal location of the driver genes (one coloured arrow per gene). (*c*) Number of mutations per nucleotide found in the entire genome (left) driver gene mutations (right).

Among the driver genes, cases showed that the most frequently affected genes in patients were *TP53* (26%), *EGFR* (22%), *ATRX/PIK3CA* (approx. 10%) and *IDH1/MKi67* (approx. 5%) (electronic supplementary material, figure S1). For an overall view of GBM aberrations, the distribution of the total mutated genes and their DNA changes was determined using the relative percentage of gene mutations according to the total length (base pairs) of each chromosome. The highest rate was found in chromosomes 19, 17 and 11, and the lowest levels were found in chromosomes 18, 13 and Y (figure 2*a*). Chromosome 1, which contains the highest number of coding genes (2076), showed a lower percentage of mutations than chromosome 17, which contains less than 60% the number of genes (1209). *TP53 (*17p13.1), which suffers from a broad amount of mutations, and *GFAP* (17q21.31), two of the most commonly used genomic markers for GBM, are found in this chromosome (figure 2*b*). Among all mutations, 95% substitutions, 3% deletions and 2% insertions were identified (figure 2*a*).

A comparison was done to determine whether the relative abundance of the types of DNA changes present in driver genes was similar to that of the whole genome. This revealed that the base substitutions were the highest both in driver genes and in the whole genome and that the nucleotide G-C change the most common (figure 2*c* and data not shown). However, mutations in the driver genes displayed a higher number of deletions and insertions than the whole genome.

**Figure 3.**
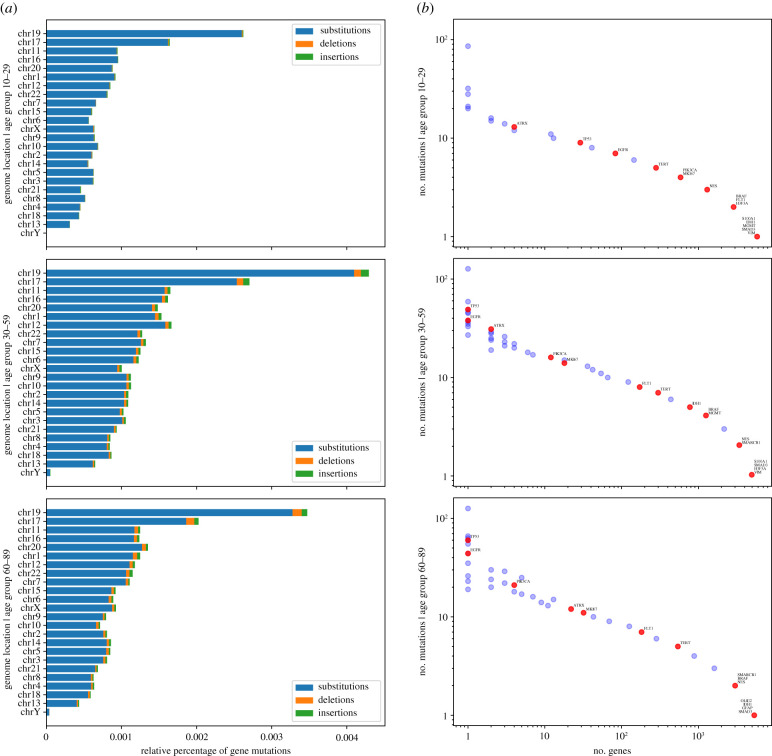
Genome location and percentage of gene mutations according to patient age subgroup. (*a*) Relative percentage of gene mutations per chromosome according to patient age: 10–29 (top), 30–59 (centre) and 60–89 years (bottom). Mutations are classified according to their type: substitutions (blue), deletions (orange) and insertions (green). (*b*) *X*-axis shows the number of genes sharing the same number of mutations, shown in *Y*-axis, grouped by patient age (same as in (*a*)); groups of genes that share the same number of mutations with driver genes represented as red dots. Data obtained from the TCGA-GBM project.

The genomic location and frequency of mutations were determined according to the patient age subgroup. Chromosomes 19, 17, 11 and 16 had the highest percentage of mutations in all subgroups. However, some chromosomes, such as 6 and 18, showed different patterns according to patient age. Regarding mutation types, substitutions were the highest in all patients, but an increase of deletions and insertions was found according to patient age (figure 3*a*). We also observed that mutations in the driver genes reflect the parallel distribution of the genome-wide mutations (figure 3*b*), as is the case in other cancers [18]. However, the frequency of mutations varies according to age group, highlighting the different mutational behaviour of driver genes in the young subgroup. In particular, *TP53* and *EGFR,* which are shown to be the most mutated genes in the adult and elderly subgroups, are not so in the young subgroup, where *ATRX* is the most affected driver gene. Among other DNA repair and chromatin remodelling genes, the mutation frequency behaviour of *IDH1*, and *MGMT* increases at 30 years of age and decreases at 60 years (figure 3*b*). When analysing these mutations in more detail, we observed that most of the mutations in all subgroups are substitutions: 91% in young, 80% in adults and 87% in the elderly.

Summarizing, the *TP53* tumour suppressor gene was found to have the highest frequency of mutations among all patient groups. For *SMARCB1*, another tumour suppressor gene, we found few mutations in adult and elderly subgroups, and none for the young subgroup (figure 3*b*).

### 3.4. Phenotypic consequences of mutations on driver genes

We also studied the phenotypic consequences of each mutation, which can often cause several of them. In the case of *TP53,* for example, a single mutation affects its 27 transcripts, causing consequences of different types. The missense variant consequence appears to be by far the most abundant, representing 47% of all consequences elicited by somatic mutations. Downstream and upstream gene variants, frameshift and intron variants, and stop gain, represent 35% of the consequences caused by mutations, and the remaining percentage is distributed among all other consequences.

Then, we focused on analysing the biological relevance of mutations on the driver genes. Polymorphism Phenotyping (polyphen) helps to predict the functional significance of an allele replacement from its features by a Naive Bayes classifier [19]. The polyphen impact reported in TCGA is a prediction of a mutation consequence being probably damaging, possibly damaging, or benign. Therefore, we used this data to indicate the possible impact of the consequence types on the function of the proteins encoded by the driver genes. As we found that polyphen impact was mainly reported for the missense variant consequence, we focused on the possible impact of amino acid substitutions.

**Figure 4.**
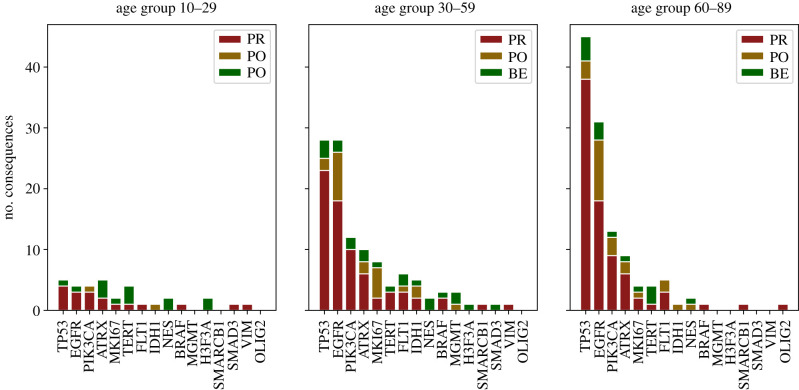
Distribution of protein phenotype impact of mutation consequences of missense variants in driver genes grouped by patient age *X*-axis depicting selected driver genes, while *Y*-axis represents the number of consequences per missense variant mutation in their corresponding canonical transcript. Consequences were classified by their protein phenotype impact regarding aggressiveness affections in probably damaging (PR, red); possibly damaging (PO, golden) and benign (BE, green). Data are shown according to the age of patients as shown on top of each panel. Data obtained from the TCGA-GBM project.

Driver gene mutations were clustered by patient age and analysed by their protein phenotype impact values. Among the driver genes, the most affected among all patient samples were *TP53*, *EGFR*, *ATRX, PIK3CA*, *IDH1* and *MKi67* (figure 4). Mutations in the tumour suppressor gene *TP53* represent one of the most common genetic lesions in cancer. In keeping with this, *TP53* was the most affected gene among the driver genes and in the whole genome, increasing abruptly with patient age, as was the case for *EGFR*. In this clinical cohort, among the DNA repair and chromatin remodelling genes, *MGMT* and *H3F3A* mutations were present only in the young and adult subgroups, with no possible negative impact on their protein functions. In *FLT1, BRAF* and *MKi67* the polyphen impact indicates damage in protein functions for the adult subgroup. *NES* and *VIM* mutations were present only among patients below 60 years of age with an unfavourable consequence in protein structure and function. For the *GFAP* and *S1001A* genes, no mutations with protein polyphen impact were found. Notably, *OLIG2* mutations with damaging impact consequences were found only in the elderly subgroup.

### 3.5. Driver gene biological pathways compared to the highest affected genes in GBM

**Figure 5.**
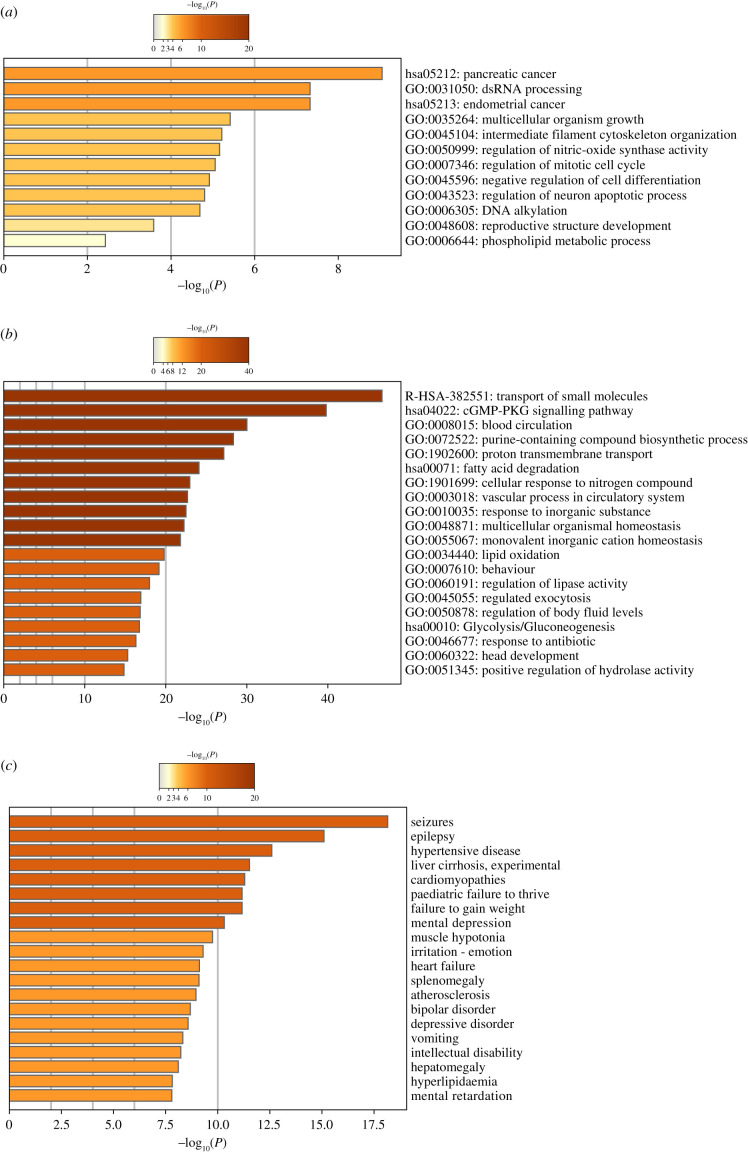
Comparison of driver gene ontology enrichment analysis with the most lesioned genes in the TCGA-GBM project. (*a*) Gene ontology enrichment and pathways for driver genes. (*b*) Heatmaps showing enrichment for most affected genes on their gene ontology and pathways (top), and DisGeNET terms (bottom). The colour key from yellow to brown indicates high to low *p*-values, respectively. Data obtained from the TCGA-GBM project.

Functional enrichment analysis was carried out for driver genes and for other genes identified with the worst protein polyphen impact. Driver genes are significantly enriched in hsa:0513 and hsa:0512 for pancreatic and endometrial cancer from the KEGG pathway (−log 10, 9.05 > −7.3), and the top GO terms include dsRNA processing, multicellular organism growth, negative regulation of cell differentiation, regulation of DNA metabolic process and regulation of neuron apoptotic process (−log10–7.3 > −4.80) (figure 5*a*). We also observed that the most affected protein phenotypes are functionally enriched in biological processes such as blood circulation, purine containing compound biosynthetic process, cellular response to nitrogen compound and vascular process in the circulatory system (−log10–30.02 < −22.68) (figure 5*b*). The biological pathways enriched were Reactome has R-HSA-382551: Transport of small molecules, (−log10–46.69), KEGG has:04022 cGMP PKG signalling pathway, has:0513 and 00071 Fatty acid degradation and has:00010 Glycolysis/gluconeogenesis pathways (−log10–39.80 > −16.74) (figure 5*b*). Those lesioned genes were linked to seizures, epilepsy, weight loss, paediatric failure to thrive, mental depression, irritation and vomiting symptoms (−log10–18 < −8.3) (figure 5*b*).

## 4. Discussion

Current clinical standard methods in neuro-oncology for GBM diagnosis consist of tumour surgery resection and biopsy followed by pathology analysis. We searched the literature over the last 15 years and found 60 clinical reports of 73 clinical cases in which patient tumour biopsy or fluid sample underwent the analysis of a combination of biomarkers which mainly consisted in *IDH1, GFAP, MKi67* and *MGMT* coupled in sets with more than two and up to 11 additional markers per sample for diagnosis. Molecular markers were reported for their relevance as measurable indicators of the presence and severity of GBM. Among those genes, the measures on the expression of *ATRX, MGMT, FLT1, GFAP, MKi67, NES, OLIG2, S1001A, VIM*, *PIK3CA*, as well as the genetic analysis of driver mutation events in *BRAF, H3F3A, TERT, EGFR, IDH1, SMAD3, TP53* and *SMARCB1* were highlighted from our literature search strategy. We searched among clinical results for a pattern of biomarker behaviour in the analysed samples with unsuccessful results. Aware of inter-tumour molecular heterogeneity as a significant challenge, and due to the remarkable importance of driver genes for the routine clinical role, we delved into their biological behaviour. A compendium of summarized findings of driver genes is shown in electronic supplementary material, file 2.

GBM inter-tumour heterogeneity allows molecular subclassification based on genomic profiling. This is also affected by intra-tumour heterogeneity, originating from two proposed mechanisms, clonal evolution and cancer stem cells. Clonal evolution is the process by which a single cell undergoes reiterative genetic changes which allows it to evolve and disseminate, forming a tumour [20]. By contrast, cancer cells in GBM could possess different stemness according to their cellular ontology, being a direct transformation from a normal stem cell or a reprogramming process from a cancer stem cell with less proliferative or differentiation capacity [17]. The GBM tumour consists of a core region of high cell proliferation and inflammation, delimited by a margin between the tumour tissue and the normal brain cells, and then the peritumoral brain zone mainly composed of normal tissue with some infiltrative and isolated tumour cells [16].

Based on a multi-omic analysis, we herein describe the heterogeneity of GBM at the transcriptional and the genomic levels, with an emphasis on tumorigenesis driver genes currently used in the clinic as molecular markers. Altogether, our results suggest that a combination of these biomarkers would provide a multidimensional approach for a better diagnosis and GBM subtype molecular classification for patient prognosis. Besides, our studies for gene expression and somatic mutations will provide information on the heterogeneity of primary GBM types due to their clinical relevance.

Our transcriptomics analysis from mRNA expression data agrees with previous reports with respect to the mesenchymal subtype. This subtype is characterized by its poor prognosis, stem cell biomarkers, angiogenesis and prominent radio- and chemoresistance. From the 18 genes analysed, we found upregulation of *MGMT,* which may be related to its own promoter's unmethylated status frequently observed in this GBM subtype and related to temozolomide treatment resistance and short patient survival [21]. In our analysis, this expression profile was conserved during adult and elderly life stages.

Furthermore, the downregulation of *ATRX, H3F3A* and *EGFR* was observed. *ATRX* encodes an adaptor protein that contributes to the Methyl-CpG binding protein 2 (*MeCP2*)-mediated pericentric heterochromatin organization, which is very important for neural differentiation [22]; thus, downregulation of this gene might be expected in cells of a less differentiated subtype with more stemness such as the mesenchymal GBM subtype. The opposite, upregulated behaviour, was observed in the proneural subtype, which has less stemness and more characteristics of differentiated cells. Another chromatin remodeller, *H3F3A,* whose driver mutations HK27M and G34R induce dysfunction of Polycomb repressive complex 2 (*PRC2*) and dramatic alterations of gene expression [23,24], may contribute to high alterations in profile expression for mesenchymal GBM subtype. *EGFR*, which is perhaps one of the best-characterized molecules in primary GBM [25], showed a downregulation in mesenchymal and proneural subtypes, but a clear upregulation in the classical GBM subtype. This behaviour is conserved across all age groups and strikingly marked for the elderly population. This expression profile could be dependent on mesenchymal GBM increased mutation rates, which may play a feedback role in downregulating *EGFR* gene expression. The coexistence of mutations in critical molecules from downstream *EGFR* signalling such as Ras or *PTEN*, which maintain active signalling without a ligand to the receptor, could play a role as an alternative mechanism.

We observed other genes with striking profile expression, including *NES, VIM* and *TP53*, with upregulation behaviour. *NES* and *VIM* encode the intermediate filament proteins Nestin and Vimentin. Vimentin is expressed mainly in mesenchymal cell types, while Nestin mainly in neural stem and progenitor cells in the central nervous system [26]. These proteins function not only as part of the cytoskeleton, but also impact several key cellular processes such as proliferation, death, migration and invasiveness [26]. Our analysis showed that *VIM* is upregulated in both mesenchymal and classical GBM subtypes and *NES* only in the classical subtype. This pattern may be related to the ontogenesis of these tumours and suggest the transition state for classical GBM to a possible mesenchymal GBM, but with a neural stem cell marker remaining.

The proneural GBM subtype showed upregulation of *MKi67* and *OLIG2. MKi67* encodes the DNA binding protein Ki-67 and is widely used as a proliferation marker as it participates in chromosome motility and chromatin organization during the cell cycle [27]. *OLIG2* encodes a central nervous system transcription factor that plays an essential role in the proliferation of oligodendrocyte precursors and their differentiation [28]. *OLIG2* also showed downregulation in classical and mesenchymal GBM subtypes. Therefore, these expression patterns support the idea that the proneural GBM subtype arises from central nervous system progenitors with fewer stemness properties but with proliferative capacity.

Our analysis in the expression profile for the 18 driver genes supports the GBM ontogenesis hypothesis from Celiku *et al*. [17], which proposes that proneural subtypes can be generated from neural progenitors, and these cells may gain somatic mutations to become classical and consecutively mesenchymal subtypes. It is also possible that classical or mesenchymal subtypes originate from central nervous system progenitors with high stemness.

In this study, we found that all driver genes have reported mutations in GBM patients. However, genes that are significantly mutated and that display multiple biological consequences include *TP53, ATRX, PIK3CA* and *EGFR.* Abnormalities of *TP53* have been the most extensively investigated genetic variations found in more than 50% of human tumours [29]. Contrary to other reports where *TP53* mutations are more related to paediatric tumours [30], we found an increasing behaviour from the young to elderly subgroups. The same behaviour is observed for genes *ATRX, PIK3CA* and *EGFR.* However, *TP53* and *EGFR* were found to be the most mutated genes in adult and elderly subgroups, while these mutational behaviour changes in the young subgroup, in which *ATRX* is the most affected gene (figure 4*b*).

Impairment of DNA repair is expected to increase the overall frequency of mutations and, hence, the likelihood of cancer-causing mutations. In comparison to other studies in which *ATRX* was found to be mutated only rarely in adult primary GBM, but frequently found in younger adults with lower-grade glioma (WHO grade II/III) [31], we found a high frequency at 30 years that decreases in elderly patients. A similar behaviour was observed for the DNA repair and chromatin remodelling genes *IDH1* and *MGMT*.

Additionally, *NES* and *VIM* mutations were absent in the elderly subgroup and are present only in patients below 60 years of age with an unfavourable consequence in protein structure and function. By contrast, *OLIG2* mutations with negative impact consequences were found only in the elderly patient subgroup.

Some driver mutations on key genes have been pivotal for the diagnosis and prognosis of GBM patients. We focused particularly on the effects of mutations with non-synonymous changes, also called missense mutations, which alter the codons so that they specify different amino acids during protein synthesis (electronic supplementary material, figure S2), and carried out a comparison of GO enriched terms of the selected driver genes with those identified with a higher probability of damaging consequences. Similar in lethality and aggressiveness to GBM, pancreatic cancer is a solid tumour difficult to treat and often fatal, characterized by the absence of early symptoms. Driver genes of tumorigenesis shared between GMB and the hsa:0513 pancreatic cancer pathway are *BRAF*, *EGFR*, *SMAD3*, *PIK3CA*, *TP53*, *IDH1*, *TERT*, *VIM*, *ATRX* and *GFAP.* Owing to their high proliferative condition, cancer cells have an increased demand for nutrients. As a mechanism, tumours alter their metabolism to feed their extensive anabolic requirements having a uniquely high demand for amino acids. Accordingly, upregulation of selective amino acid transporters has been reported [32]. R-HSA-382551 transport of small molecules pathway is involved in the regulation and movement of small molecules across plasma membranes and between cellular organelle compartments within cells. Our functional enrichment analysis on highly affected proteins shows a significant abundance of the solute carrier (SLC) superfamily related to this pathway. Examples of highly lesioned solute carrier proteins found are SLC1A6 (with 10 probably damaging (PR) consequences), SLC5A7 (6: PR), SLC9A2 (6: PR) and SLC6A19 (4: PR). In pancreatic cancer, the clinical potential of an amino acid transporter SLC6A14 as a drug target has been recently reported [32]. We also found that highly affected pathways such as blood circulation and vascular processes in the circulatory system are consistent with alterations in angiogenesis in GBM. We also identified a link of the lesioned proteins to seizures, epilepsy, weight loss, paediatric failure to thrive, mental depression, irritation and vomiting, among other symptoms that are in agreement with those reported in the clinical cases reviewed.

Efforts have been made for the identification of relevant biomarkers to assess GBM progression by targeting genes with the highest density of missense mutations. For example, tumours with the *BRAF* V600E mutation tend to be more severe. This somatic mutation prevents the *Braf* protein from controlling cell proliferation (electronic supplementary material, figure S3), which has been reported in the TCGA database, appearing at all ages but more frequently in elderly patients.

*TP53* mutations were predominantly point mutations, which lead to amino acid substitutions in the DNA binding domain (DBD). The substitution of arginine residues within the DBD, such as R175, R248 and R273, was reported in other studies and was also found in GBM patients [33]. However, this was not the most abundant amino acid substitution, being G105R, S127Y, P152S and V157G, examples of some amino acid changes abundantly reported in the TCGA cohort.

The most cited biomarker for diagnosis *IDH1* R132H has also been reported in the TCGA database as a mutation in all age subgroups with a negative polyphen impact [3]. On the other hand, the H3K27M mutation that has been highly linked to paediatric thalamic gliomas and is associated with a worse prognosis than low-grade tumours were not found in the TCGA cohort, which is the case of other biomarkers used in clinical studies, such as H3G34R, H3G34N, *EGFR* R776C, and the *TERT* promoter mutations C228T and C250T [23,24,31].

To understand better the behaviour of mutations in young patients, we analysed genes that are involved in GBM with the worst polyphen impact consequences and analysed the transcription factors that regulate them. Our results showed that the young subgroup behaves differently, as genes that are mutated are regulated by different transcription factors (TFs). Moreover, the TFs that regulate genes with mutations in the young subgroup share almost no TFs with adult and elderly subgroups. This might explain why the young subgroup has a divergent behaviour in comparison with the other subgroups. On the other hand, the adult and elderly subgroups share most of the biological pathways, while microtubule cytoskeleton organization, regulation of microtubule-based process, adenylate cyclase-inhibiting G protein-coupled glutamate receptor signalling among others are GO terms unique for the adult subgroup, while protein–protein interactions at synapses, regulation of cyclase activity and carbohydrate digestion and absorption are unique functional terms for the elderly subgroup. In particular, genes with mutations with a negative polyphen impact in the 10–29-year-old subgroup share fewer identities with the 60–89-year-old subgroup (electronic supplementary material, figure S3).

It is surprising that among all the TCGA data reported for GBM, several mutations that are defined as biomarkers could not be found. The absence of a clearly defined and concordant pattern between clinical, transcriptomics and mutational dynamics studies, support the idea of outstanding heterogeneity in GBM. Despite the high abundance of somatic aberrations in GBM tumours, only a select few have been associated with clinical relevance and are currently used as biomarkers. No single mutation has been identified to trigger a particular type of GBM tumour. The intra- and inter-tumour heterogeneity of GBM has revealed its ‘multiforme’ nature not only at its morphologic and phenotypic levels but also on its genotype.

Furthermore, the relationship between genetic alterations and gene expression at the mRNA level is not always linear. The interplay between distant genetic interactions and epigenetic changes also has a significant impact on the expression of specific genes. Hence, the selection of the most commonly mutated and amplified genes as therapeutic targets may not be sufficient. Our results showed that the link of markers and profile expression with their phenotypic alterations is more complex than previously thought. With this analysis, however, we expect to contribute to the construction of a panel of driver genes to delineate better the intra- and inter-tumour heterogeneity for a more accurate diagnosis. To achieve this objective, it is crucial to analyse the raw data for other key molecules involved in the mechanisms that drive the balance between proliferation and differentiation in the stem and cell precursors for the central nervous system.

**Figure 6.**
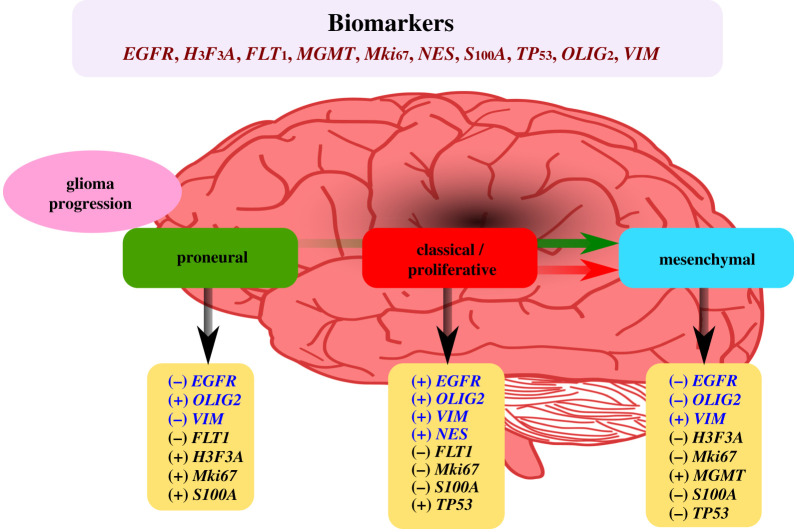
Proposed biomarker panel to determine the GBM molecular subtype. Summarized gene expression analysis showed that the altered expression pattern for the 18 driver genes supports the GBM glioma progression model, which proposes that proneural subtypes can be generated from neural progenitors, and these cells may gain somatic mutations to become classical and consecutively mesenchymal subtypes. Combination of the gene biomarkers *EGFR, H3F3A, FLT1, MGMT, Mki67, NES, S100A, TP53, OLIG2 and VIM* could help to determine the GBM molecular subtype for patient prognosis. *EGFR, NES, OLIG2 and VIM (highlighted in dark blue*) show a strong differential expression pattern.

Currently, expression levels of *ATRX*, *MGMT*, *FLT1*, *GFAP*, *MKi67*, *NES*, *OLIG2*, *S1001A*, *VIM* and *PIK3CA* are used in the clinic for patient GBM diagnosis and prognosis. Our results indicate that the biomarker set integrated by *EGFR*, *H3F3A*, *FLT1*, *MGMT*, *MKi67*, *NES*, *S100A*, *TP53*, *OLIG2 *and* VIM* genes could be a strong combination to determine the GBM molecular subtype (figure 6). For example, the mesenchymal subtype, known as the most aggressive GBM, showed overexpression of *MGMT* and *VIM*, and the repression of *EGFR*, *H3F3A*, *OLIG2*, *S100A* and *TP53.* On the other hand, while overexpression of *EGFR*, *NES*, *VIM* and *TP53* was characteristic of the proliferative or classical subtype, concomitant overexpression of *MKI67* and *OLIG2* could be more favourable prognosis owing to their association with the less aggressive proneural subtype. Recently, Teo *et al.* based on TCGA data used a set of 1500 genes from GBM subtypes across Caucasian, Korean and Chinese populations [34]. In comparison, from our selected driver genes *EGFR*, *IDH1*, *MKI67*, *NES*, *S100A1* and *VIM* were reported to be differentially expressed and overlapped with the three datasets of TCGA-GBM populations samples. Moreover, *EGFR, NES* and *S100A1* are among their selected 500 genes used for the classification of the three GBM subtypes. Furthermore, in accordance with their study we also identified that *EGFR* presents subtype specificity. In addition, our study suggests that *NES*, *OLIG2* and *VIM* are also subtype specific genes. Altogether, our findings indicate that *EGFR*, *NES*, *OLIG2* and *VIM* genes represent an outstanding selection of biomarkers for patient prognosis, since a remarkable differential pattern from the combination of them was revealed by our transcriptomic analysis (figure 6). Further clinical trials with patient samples for expression analysis, together with the development and application of gene expression-based classifier algorithms for molecular subtypes testing the above-mentioned biomarkers could provide confirmatory evidence for their clinical potential.

## 5. Conclusion

GBM is a highly heterogeneous cancer that consists of multiple molecular alterations. Despite the vertiginous advances in the clinical medical area, the prognosis of patients continues to be unfavourable, with an average survival of less than 1 year. The differential molecular characteristics of histologically similar tumours make it difficult to predict clinical outcomes and select optimal treatment strategies. Given the heterogeneity of GBM and the multitude of factors that influence disease progression, general clinical characteristics are insufficient to predict individual prognosis and survival accurately. In the clinical routine, a combination of biomarkers is necessary for differential diagnosis and prognosis being *IDH1*, *GFAP*, *Mki67* and *MGMT* the most reported ones. The inter-tumour molecular heterogeneity remains the hardest challenge in neuro-oncology practice. In our study, the expression profiles of those markers revealed a consistent link with the progression model for GBM tumour ontogenesis, supporting that tumours display a unique behaviour and that ‘personalized’ treatment must be required for each molecular subtype. Our results indicate that a combination of the biomarker genes *EGFR*, *NES*, *OLIG2* and *VIM* could be a strong set to determine the GBM molecular subtype for patient prognosis. Notably, the frequency of mutations varies according to age group, highlighting the different mutational behaviour of driver genes in the young subgroup. In particular, *TP53* and *EGFR,* which are the most mutated genes in the adult and elderly subgroups, are not mutated in the young subgroup, in which *ATRX* is the most affected driver gene. Besides, a unique distribution of somatic mutations was found for the young and adult populations, particularly for the genes related to DNA repair and chromatin remodelling *ATRX*, *MGMT* and *IDH1*. We also identified regulatory and biological pathway behaviours that varied with age which could serve as a basis for further analysis in the journey of the development of improved therapy for patients suffering from this disease.

## Data accessibility

Data source for the gene expression analysis Glioblastoma BioDiscovery Portal (GBM-BioDP) https://gbm-biodp.nci.nih.gov. Data source for somatic mutations of GBM https://portal.gdc.cancer.gov/. Github Repository for codes Python scripts (https://github.com/kap8416/GLIOBLASTOMA-MULTIFORME-A-META-ANALYSIS-OFDRIVER-GENES-CURRENT-DIAGNOSIS-AND-TUMOR-HETEROGENEITY).
